# Mandibular Mucormycosis Following Severe COVID-19 in a Patient With Chronic Drug Abuse: A Case Report

**DOI:** 10.7759/cureus.95542

**Published:** 2025-10-27

**Authors:** Oscar Arturo Benitez-Cárdenas, Miguel Ángel Noyola-Frías, Elhi Manuel Torres-Hernández, Ricardo Martínez-Rider, Marlen Vitales-Noyola

**Affiliations:** 1 Oral and Maxillofacial Surgery Specialty, Faculty of Dentistry, Autonomous University of San Luis Potosi, San Luis Potosí, MEX; 2 Service of Oral and Maxillofacial Surgery, Regional High Specialty Hospital “Dr. Ignacio Morones Prieto”, San Luis Potosí, MEX; 3 Endodontics Posgraduation Program, Faculty of Dentistry, Autonomous University of San Luis Potosi, San Luis Potosí, MEX

**Keywords:** covid-19, drug addict, mandibulectomy, maxillectomy, mucorals, mucormycosis

## Abstract

Mucormycosis is an infection caused by mucoral fungi, particularly the genera *Rhizopus, Rhizomucor,* and *Mucor*. It leads to invasive necrotic lesions primarily in the nose and palate, manifesting symptoms such as pain, fever, orbital cellulitis, proptosis, and purulent rhinorrhea, among others. The predominant manifestations of this infection include rhino-orbital-brain and pulmonary forms, which occur predominantly in individuals with immunosuppression or diabetes. Mucormycosis is an uncommon pathology associated with a high mortality rate. Standard treatment involves the use of antimycotics and surgical intervention to remove necrotic tissue. However, even with appropriate treatment, the prognosis remains poor, and mortality continues to be substantial. This report presents the case of a 53-year-old male diagnosed with mucormycosis following severe coronavirus disease 2019 (COVID-19). The patient had a 40-year history of drug addiction and underwent maxillectomy and mandibulectomy, along with extended treatment with antimycotics, resulting in a favorable recovery. One year post-follow-up, the patient exhibits no clinical or imaging evidence of infection and uses a palatine obturator. The report describes a rare case of mandibular mucormycosis temporally associated with severe COVID-19, where virus-related immunosuppression (and its treatment) likely facilitated fungal invasion.

## Introduction

Mucormycosis is an uncommon but severe fungal infection primarily affecting the head and neck region, typically occurring in immunocompromised individuals [1]. The etiological agents belong to the order *Mucorales*, which includes the genera *Rhizopus*, *Rhizomucor*, and *Mucor*. These fungi act as opportunistic pathogens capable of causing life-threatening disease in humans and other species [2]. Although considered rare, mucormycosis has been increasingly reported worldwide [3]. In Mexico, reliable epidemiological data are lacking, as mucormycosis is not a notifiable disease within the national health surveillance system. This underreporting limits the understanding of its true incidence and hinders the development of effective public health strategies. The clinical presentation of mucormycosis varies depending on the affected tissue or organ.

The most common forms include rhino-orbital, cerebral, and pulmonary involvement, while less frequent manifestations occur in the palate, gastrointestinal tract, skin, and kidneys. The fungus typically enters through inhalation or skin disruption, spreading hematogenously or locally [4-6]. Characteristic signs and symptoms include ocular proptosis, nasal crusting, fever, headache, facial erythema, sinus congestion, orbital cellulitis, purulent rhinorrhea, ophthalmoplegia, visual impairment, and mucosal necrosis. In advanced cases, central nervous system involvement may lead to cavernous sinus thrombosis, seizures, aphasia, or hemiplegia.

Diagnosis is based on clinical findings and confirmed through histopathological and microbiological analysis. Management requires prompt antifungal therapy, most commonly intravenous amphotericin B, and surgical debridement of necrotic tissue; however, mortality rates remain high despite aggressive treatment [6]. The main predisposing factors include uncontrolled diabetes and immunosuppressive states, particularly diabetic ketoacidosis, which carries a high mortality rate. Other contributing conditions comprise hematologic malignancies, organ transplantation, intravenous drug use, trauma, AIDS, leukemia, malnutrition, and prolonged corticosteroid or immunosuppressive therapy, all of which facilitate fungal invasion and dissemination [7,8].

Since its emergence in late 2019, the coronavirus disease 2019 (COVID-19) pandemic caused by severe acute respiratory syndrome coronavirus 2 (SARS‑CoV‑2) has had a profound global impact. Beyond its respiratory manifestations, COVID-19 has been associated with various opportunistic infections, with mucormycosis emerging as a significant concern. A sharp rise in cases was observed during the pandemic, particularly in developing countries, with India reporting one of the highest incidence rates worldwide [9,10]. In 2021, Indian health authorities declared an epidemic of mucormycosis, noting that nearly 90% of cases occurred in COVID-19-positive patients [11]. This surge was attributed to uncontrolled diabetes and extensive corticosteroid use during COVID-19 treatment, both of which create favorable conditions for fungal proliferation [11].

Although official data are still lacking in Mexico, recent clinical observations indicate a rising number of post-COVID mucormycosis cases, especially in the state of San Luis Potosí. This emerging trend highlights the need for enhanced surveillance and further research to better understand its local epidemiological impact. The present report describes a male patient who developed mandibular mucormycosis following severe COVID-19 infection. He underwent surgical debridement and antifungal therapy, achieving a favorable clinical outcome.

## Case presentation

**Figure 1:**
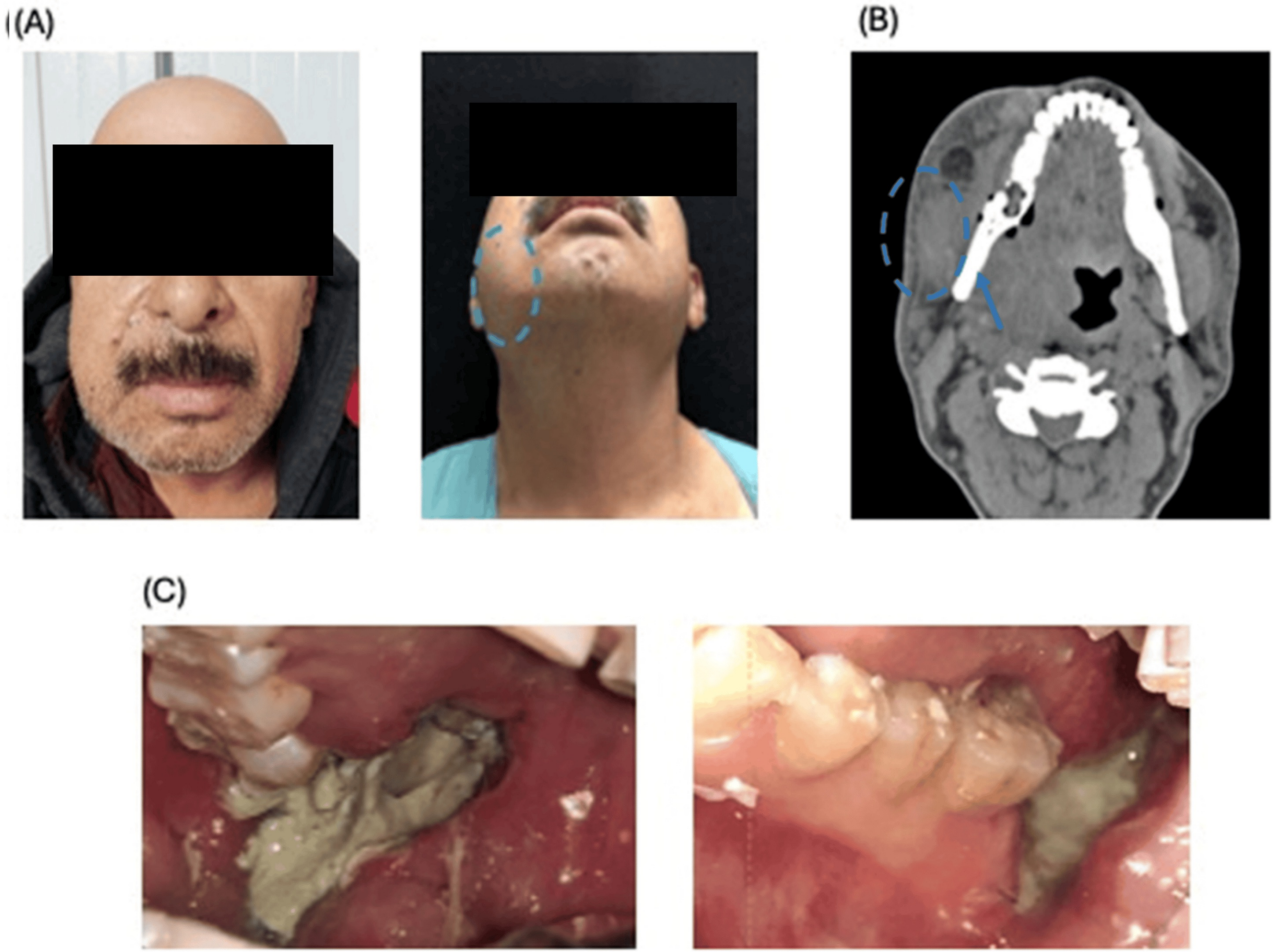
Evaluation of the patient by clinical and imaging modalities A) Images of a patient exhibiting volumetric enlargement in the right oral region. B) CT of the patient. C) Regions of bone exposure and necrotic tissue CT: computed tomography

A 53-year-old male from an indigenous Mexican community, employed as a temporary laborer, presented with multiple substance-use disorders. He had a longstanding history of alcohol and tobacco use beginning at age 12 and reported weekly use of cannabis, crystal methamphetamine, and cocaine. His psychiatric history included major depressive disorder and acute stress disorder, for which he had been prescribed citalopram 20 mg and pregabalin 75 mg. He presented to the maxillofacial surgery department with dental pain localized to the right mandibular quadrant, on tooth 48, ongoing for six months, and associated with progressive swelling of the right oral region. He was started on oral clindamycin 600 mg for seven days. A CT scan and panoramic radiograph were obtained (Figure 1).

Five days later, tooth extraction was performed, and the antibiotic course was extended for several days. One week after the extraction, he returned for reassessment due to persistent swelling, continued pain, and new-onset oronasal communication. He was admitted for management of an odontogenic abscess involving the masseteric region. The oronasal and post-extraction alveolar lesions showed progressive extension with a necrotic appearance; biopsies were obtained for histopathological analysis. On examination, there was facial asymmetry over the right mandibular/buccal region with induration and tenderness, accompanied by dysphagia and rhinolalia. Intraoral evaluation revealed trismus, exposed bone with necrotic features at the post-extraction socket of tooth 48, and an abscess with oronasal communication at the soft palate extending toward the right tonsillar pillar, characterized by gray-yellow margins and halitosis.

**Figure 2:**
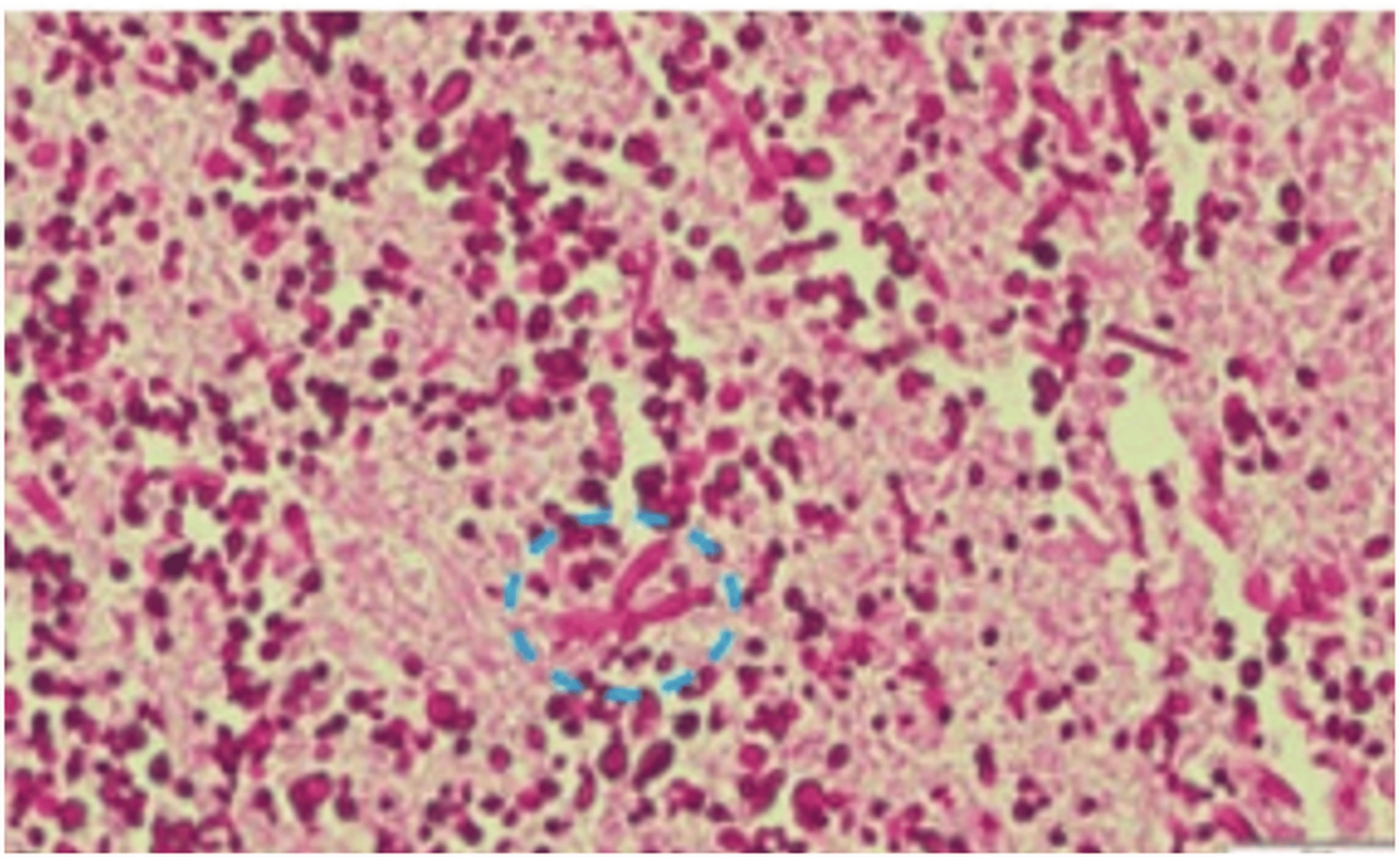
Histopathological examination A biopsy was performed on notable tissues. Corresponding staining was conducted, within the blue circle, on discernible structures consistent with hyphae, stained with per-iodic acid-Shiff (PAS), 10X magnification

A fistulous tract dissecting along the medial aspect of the mandibular ramus was identified. The patient was referred to the otorhinolaryngology service for management of the oronasal communication. A CT scan of the facial skeleton and neck, along with a panoramic radiograph, was obtained. CT demonstrated hyperdense mastoid air cells, loss of muscular insertions with overlying soft-tissue coverage, and hyperdense material involving the pterygoid plates, right maxillary tuberosity, and right maxillary sinus. There was also a mild hypodense soft-tissue expansion in the right oral region and parapharyngeal space. Incisional biopsies of the right tonsillar pillar, buccal mucosa, and soft palate were submitted for histopathology and culture. Histopathology revealed chronic inflammatory changes in soft tissues with secondary necrosis caused by primary infection with *Rhizopus spp*., concomitant *Candida krusei* co-infection was also identified. The final diagnosis was mucormycosis involving the right mandibular and palatine regions (Figure 2).

Given the diagnosis of mucormycosis and the absence of diabetes mellitus, a common predisposing factor, other risk factors were explored. The patient reported COVID-19 pneumonia approximately three months prior, with dyspnea, fever, diffuse myalgias, anosmia, and dysgeusia requiring hospitalization and supplemental oxygen. Following clinical deterioration, he was transferred to the ICU and placed on assisted mechanical ventilation (AMV). Per national protocols in Mexico during the pandemic, hospitalized patients underwent RT-PCR confirmation of SARS-CoV-2 infection, and standard regimens frequently included high-dose systemic corticosteroids. Further details regarding his inpatient course and specific therapies were unavailable, as treatment had occurred at an outside hospital and the patient could not provide additional information. After histopathological confirmation of mucormycosis, maxillofacial surgeons performed surgical management. The muco-osseous oronasal lesion and the right mandibular region were debrided by curettage, followed by a right maxillectomy (Brown classification 2B).

**Figure 3:**
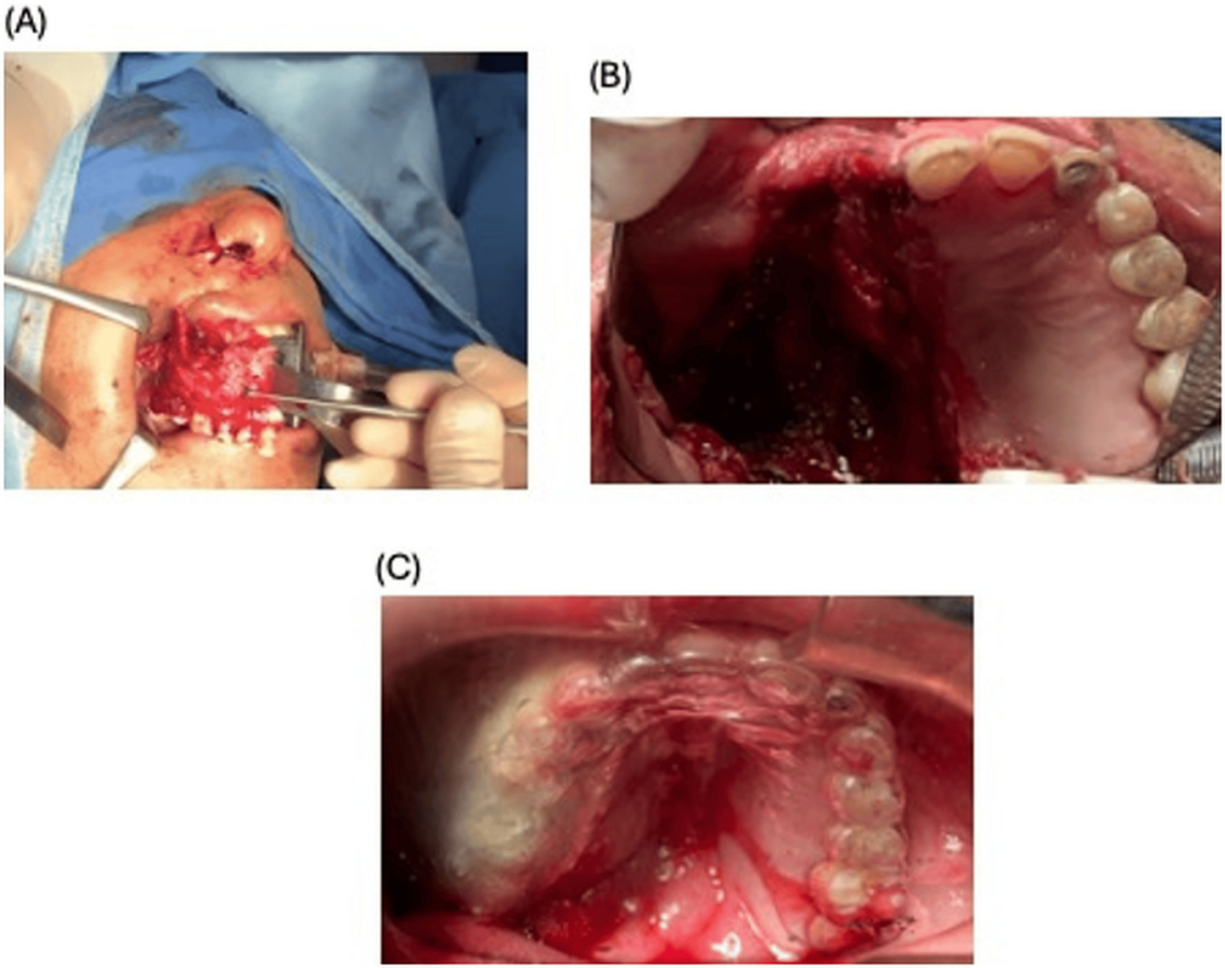
Surgical intervention A) Type 2B right-sided maxillectomy, right body marginal mandibulectomy, and necrotic tissue resection. B) Postoperative image. C) Palatine obturator positioned on the patient

A right marginal body mandibulectomy was performed with resection of necrotic tissue involving the palate, tonsillar pillar, and right lingual soft tissues (Figure 3). A palatal obturator was placed, and furocin-impregnated gauze packing was applied (Figure 3). Estimated blood loss was approximately 600 mL, and the patient required a transfusion. Balanced general anesthesia was administered via a size-8 orotracheal tube. A right subclavian central venous catheter was inserted, and local infiltration with 2% lidocaine plus epinephrine was used. A right circumvestibular intraoral approach was undertaken; osteotomies extended from the canine eminence to the pterygoid plates, with preservation of the mental foramen. Following debridement and resection of devitalized tissues (palate, tonsillar pillar, and right lingual region), the right marginal mandibulectomy was completed. Hemostasis was secured, and wounds were closed with 3-0 Vicryl sutures. The palatal obturator was left *in situ*.

The Infectious Diseases service initiated intravenous meropenem (1 g) plus amphotericin B (1 mg). Postoperatively, the patient was managed by the pain service with intravenous tramadol (100 mg). In the ensuing days, he reported severe, intermittent stabbing pain in the right hemifacial region radiating to the neck, with paroxysms lacking clear triggers. He was referred to psychiatry for suspected withdrawal syndrome, but requested voluntary discharge and did not continue treatment. The patient attended scheduled follow-ups. At the two-year review, he remained clinically well with no evidence of recurrent mucormycosis and was asymptomatic. Definitive facial reconstruction is pending due to financial constraints. To facilitate case-to-case comparison, a concise chronological summary of symptom evolution, investigations, and interventions is provided in Table 1. 

**Table 1:** Timeline of symptom progression COVID-19: coronavirus disease 2019; ICU: intensive care unit; CT: computed tomography

Time	Event/intervention	Key findings/notes
Month 6	Symptom onset	Dental pain at tooth 48, progressive right-sided oral swelling
Month 3	Severe COVID-19	Hospitalization, ICU, assisted ventilation, likely high-dose steroids
Day 0	Initial visit to the Oral Maxillofacial Surgery service	CT + panoramic radiograph, clindamycin 600 mg, 7 days
Day +5	Dental extraction	Tooth 48 removed, antibiotics extended
Day +12	Re-presentation and admission	Persistent pain/swelling; oronasal communication; multiple biopsies
Day +15	Histopathology	Mucormycosis (Rhizopus spp.) + *Candida krusei*, secondary infection
Day +16	Surgery #1	Curettage + right maxillectomy (Brown 2B)
Day +20	Surgery #2	Right marginal mandibulectomy, necrotic tissue resection, obturator placed, closure with 3-0 Vicryl
Immediate postoperative	Medical therapy	Meropenem IV 1 g + amphotericin B IV 1 mg; tramadol IV 100 mg
Early course	Interconsult	Hemifacial stabbing pain, psychiatry for withdrawal, voluntary discharge
Month +24	Follow-up	Disease-free, asymptomatic; reconstruction pending

## Discussion

The COVID-19 pandemic has led to a significant rise in the incidence of rare infectious diseases, including mucormycosis, across various regions globally. The rise in frequency of these pathologies remains partially unexplained; however, it is reasonable to infer that tissue hypoxia in COVID-19 patients contributes to the proliferation of opportunistic fungi [12]. Initially, our patient had experienced a severe case of COVID-19 during the early stages of the pandemic, when clinical manifestations were more severe, and mortality rates were considerably higher; however, these significantly declined with the introduction of vaccines and antivirals. The proliferation of mucoral fungi is influenced by several factors, including comorbidities such as uncontrolled diabetes, neutropenia, cancer, elevated serum ferritin levels, and/or the use of corticosteroids. The elevated corticosteroid doses induce immunosuppression, constituting a risk factor for mucormycosis.

The initial treatment for COVID-19 involved administering high doses of corticosteroids, and patients exhibited elevated serum ferritin levels. These interventions, while essential for managing severe respiratory symptoms, are known to compromise immune function and have been linked to an increased risk of opportunistic fungal infections such as mucormycosis [12]. The immunosuppression causes a decrease in the number and function of leukocytes, a phenomenon that was observed in these patients with COVID-19. This compromised immune state also promotes greater reproduction of these fungi, raising the likelihood of developing the most severe and fatal form of mucormycosis, which is rhinocerebral [13].

Additionally, various authors from different parts of the world have reported this association between mucormycosis and COVID-19 [7,9,10]. A major surge of COVID-19-associated mucormycosis (CAM) in India (2021) highlighted how steroid-induced hyperglycemia, diabetes, immune dysregulation, and prolonged hospitalization can enable *Mucorales* infection. While rhino-orbito-cerebral CAM predominated, mandibular involvement is rare. Our case expands the anatomical spectrum and reinforces the need for vigilance beyond sinonasal disease. Early diagnosis, prompt antifungals, and surgical debridement remain critical.

Our patient did not exhibit any comorbidities, such as diabetes or other chronic-degenerative or autoimmune diseases; however, the patient had a 40-year history of drug addiction. This condition may contribute to immunosuppression and the advancement of infectious diseases. Additionally, it is noteworthy that the patient resides in a rural community and works as a day laborer, which involves contact with soil and various animals, so this exposure may have facilitated the acquisition of the fungus. Furthermore, the habitual use of drugs via the nasal route is associated with poor hygiene practices; however, in this case, drug addiction is reported only as part of the patient’s history and is not considered a predisposing factor for mucormycosis. The most plausible driver was COVID-19-related immunosuppression (and its treatment), which likely facilitated fungal invasion.

In addition to the aforementioned factors, tissue hypoxia resulting from COVID-19 must also be considered as an additional risk factor [12]. It is important to mention that in most cases, mucormycosis appears in the first few weeks after the COVID-19 diagnosis, but in this case, the patient had a history of severe COVID-19 three months prior, which may have influenced the late onset of the fungal infection. Initially, during the pandemic and before the introduction of vaccines and antivirals for SARS-CoV-2, COVID-19 raised a significant health risk, potentially resulting in severe illness and mortality. The initial treatment protocol included high doses of steroids, AMV, and elevated oxygen volumes. Consequently, these patients likely faced an elevated risk of mucormycosis due to underlying conditions, the application of AMV, hyperglycemia, hypoxia, and the inflammatory immune response [13,14]. The patient experienced a severe case of COVID-19, which, combined with other risk factors, increased the risk of mortality from mucormycosis infection. Nonetheless, with appropriate interventions, including extended antifungal therapy and surgical procedures, the patient exhibited a favorable prognosis.

In patients with mucormycosis affecting facial structures, including the paranasal sinuses, maxilla, mandible, and orbit, the preferred surgical intervention is debridement to excise necrotic tissue. The patient underwent maxillectomy and mandibulectomy to remove necrotic tissues and bones, resulting in a favorable prognosis with this surgical intervention alongside antimicrobial therapy [15]. The spread of the infection to the oral cavity can cause necrosis of the palate in certain patients, leading to palatal perforation. In our patient, oral placement was performed following surgical treatment. Airway management in patients with mucormycosis may present challenges due to facial swelling and pain. However, in this case, airway management was simple, and the patient experienced no complications.

The pain linked to maxillofacial tissue invasion from mucormycosis can lead to restricted mouth opening in some patients. Our patient exhibited limited mouth opening, complicating physical examination; however, this issue resolved over time [15]. In the context of antimicrobial treatment, amphotericin B is the preferred antifungal for these cases, demonstrating a high efficacy against the fungus responsible for mucormycosis [16]. Several mechanisms have been proposed to explain the onset of mucormycosis in individuals with COVID-19 and diabetes, emphasizing the role of elevated serum glucose levels and ferritin levels, which are recognized for their contribution to Mucor metabolism [16]. The relationship between mucormycosis and COVID-19 is complex and is influenced by the immune response triggered by SARS-CoV-2 infection. This response is marked by a reduction in the functions of lymphocytes and neutrophils, including chemotaxis, cytokine secretion, and phagocytosis. Prolonged hospitalization is typically linked to invasive procedures, including catheters and mechanical ventilation. Both situations promote the proliferation of fungi in the environment, establishing conditions that facilitate the progression of this aggressive disease [16]. The patient, diagnosed with mucormycosis, was infected with COVID-19 and simultaneously presented multiple substance abuse disorders.

Recent reports from India and other South Asian countries have highlighted that CAM is no longer a rare occurrence but a significant post-pandemic complication. The combination of steroid-induced hyperglycemia, diabetes, and virus-related immune dysregulation has been recognized as the main predisposing triad for CAM development. These findings have led to the establishment of standardized treatment protocols emphasizing early diagnosis, surgical debridement, and liposomal amphotericin B therapy [17-19]. Liposomal amphotericin B remains the first-line treatment, followed by step-down therapy with posaconazole or isavuconazole [20]. In maxillofacial cases, surgical planning is often complex due to the need to balance radical debridement with preservation of function and facial symmetry.

In our patient, the extent of mandibular involvement made reconstruction challenging, necessitating a staged approach following infection control. Multidisciplinary collaboration was essential to optimize both therapeutic outcomes and postoperative recovery. In summary, several studies have reported a marked rise in mucormycosis among COVID-19 patients, underscoring the severity of this coinfection and its association with factors such as corticosteroid use and immunosuppression. The outbreaks observed across various regions highlight the importance of a vigilant clinical approach and early diagnosis to improve patient outcomes.

## Conclusions

We discussed a case of mandibular mucormycosis in a non-diabetic patient, occurring in temporal association with severe COVID-19, where immunosuppression related to the virus and its treatment likely contributed to the infection. Drug use was noted in the patient’s history, but was not considered a predisposing factor. In light of recent increases in post-COVID fungal infections, clinicians should maintain a high index of suspicion and prioritize early diagnosis, surgical debridement, and antifungal therapy.
